# Toxicological Assessment of Inhaled Nanoparticles: Role of *in Vivo*, *ex Vivo*, *in Vitro*, and *in Silico* Studies

**DOI:** 10.3390/ijms15034795

**Published:** 2014-03-18

**Authors:** Eleonore Fröhlich, Sharareh Salar-Behzadi

**Affiliations:** 1 Center for Medical Research, Medical University of Graz, Stiftingtalstr. 24, Graz A-8010, Austria; 2 Research Center Pharmaceutical Engineering GmbH, Inffeldgasse 13/II, Graz A-8010, Austria; E-Mail: sharareh.salar-behzadi@rcpe.at

**Keywords:** cell culture, air-liquid interface, inhalation exposure models, species differences, *in silico* modeling

## Abstract

The alveolar epithelium of the lung is by far the most permeable epithelial barrier of the human body. The risk for adverse effects by inhaled nanoparticles (NPs) depends on their hazard (negative action on cells and organism) and on exposure (concentration in the inhaled air and pattern of deposition in the lung). With the development of advanced *in vitro* models, not only *in vivo*, but also cellular studies can be used for toxicological testing. Advanced *in vitro* studies use combinations of cells cultured in the air-liquid interface. These cultures are useful for particle uptake and mechanistic studies. Whole-body, nose-only, and lung-only exposures of animals could help to determine retention of NPs in the body. Both approaches also have their limitations; cellular studies cannot mimic the entire organism and data obtained by inhalation exposure of rodents have limitations due to differences in the respiratory system from that of humans. Simulation programs for lung deposition in humans could help to determine the relevance of the biological findings. Combination of biological data generated in different biological models and *in silico* modeling appears suitable for a realistic estimation of potential risks by inhalation exposure to NPs.

## 1. Introduction

Nanoparticles (NPs) are defined as objects measuring ≤100 nm in one dimension [1]. In pharmacy and medicine also larger particles (up to 1 μm) are included in this definition. NPs improve quality, lifetime, appearance, storage, *etc*., of many industrial, consumer, and medical products. As a consequence of the increased production of NPs, human exposure to unintentionally produced NPs (air pollution, byproducts during production) and to engineered NPs has increased markedly in the last decade. NPs can be found in soil, water, food, and air and may be taken up by humans by oral, dermal, or inhalation route.

**Figure 1.**
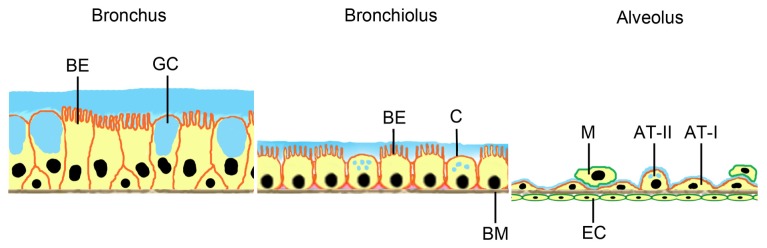
Barriers for particle uptake by the respiratory system. Surfaces of larger (conducting) airways are mainly covered by bronchial epithelial cells with cilia (BE) and mucus (blue) producing goblet cells (GC). In bronchioli, bronchial epithelial cells and mucus producing cells (Clara cells, C) are found. All epithelial cells reside on a basement membrane (BM). The air-blood barrier at the alveolus consists of alveolar epithelial cells type I (AT-I) and surfactant-producing AT-II cells. Alveolar macrophages (M) migrate on top of the alveolar epithelial cell layer. On the other side of the basement membrane endothelial cells (EC) of capillaries are located.

Internal and external surfaces of the human body are covered by epithelia to prevent the uncontrolled penetration of foreign substances. Although all epithelia reside on a basal membrane with connective tissue beneath, epithelial cells by formation of intercellular junctions, cellular differentiation (e.g., keratinization) or secretion of mucus represent the main barrier for entry in the body. To enter systemic circulation, the substances have, in addition, to cross the endothelium of blood vessels located in the connective tissue. Permeability of the endothelium, in general, is higher than that of the epithelia covering skin and mucosae. Relevant epithelial barriers are mucus producing bronchial cells in the conducting airways. In the deep lung, where the air-blood barrier is located, alveolar cells covered by surfactant regulate the entrance of foreign substance into the body (Figure 1). This barrier, being only 0.1–0.2 μm thick, is the most permeable barrier of the human body.

NPs are small enough to reach the deep lung and get in contact with air-blood barrier, while larger particles (>5 μm) are trapped in the upper airways, where the epithelial lining is thicker and cells are covered with protective mucus (Figure 1, comparison bronchus/bronchiolus/alveolus). Epidemiological studies showed that exposure of humans to ultrafine particles (<2.5 μm) in the air increased pulmonary morbidity and mortality (e.g., [2–7]). Lung damage presenting as pulmonary fibrosis and pleural granuloma formation was reported in several workers 5–13 weeks after exposure to polyacrylate NPs [8]. Particles were detected in cytoplasm and nucleus of pneumocytes and mesothelial cells. Animal studies revealed that NPs at equivalent mass doses cause inflammation and cross the alveolar barrier in much higher numbers than larger particles [9].

To evaluate health risk to the general population and to workers by inhalation of airborne NPs, hazard and exposure have to be taken into account. Exposure depends on the concentration of NPs in the air, to which the individual is exposed, and on the likelihood that these particles will deposit in the respiratory tract. Particle numbers and particle size distributions are parameters used to quantify NP exposure in the air. Numerous studies addressed adverse effects of airborne NPs using *in vitro* and *in vivo* systems to compare biological effects of micro- and nanoparticles and identify potential NP-specific action [10]. Problems with the generation of these exposure data, particularly the lack of differentiation between background and NPs, have been addressed in several reviews (e.g., [11]). Lack of sensitivity of the measurement devices and problems in characterization of the NPs, lack of a systematic approach, harmonization and standardization of measurements, *etc*., complicate the evaluation of workers’ exposure to NPs [10].

Particle in the nasal cavity can use two uptake routes. They can cross the respiratory epithelium and reach the underlying blood vessels. Alternatively, NPs can be adsorbed through the olfactory epithelium, are transported along the olfactory bulb and reach the brain. A particle deposition model indicated that about 11% of manganese oxide NPs deposited on the rat olfactory mucosa reached the olfactory bulb [12]. Similar data were also obtained for translocation of ^13^C NPs in rats and silver-coated gold NPs in squirrel monkeys [13,14]. The relevance of this uptake route for humans is expected to be lower because the olfactory mucosa represents 5% of the total nasal mucosa in humans but 50% in rats. In addition, adult humans in contrast to newborns and to many other mammals (e.g., rodents, rabbits, horses) are not obligatory nose breathers but nose/oral breathers [15].

**Figure 2.**
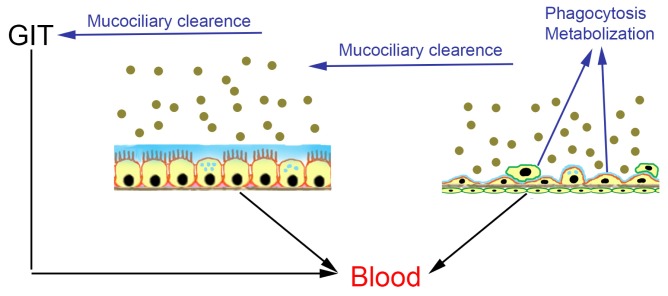
Fate of inhaled nanoparticles in conducting airways (bronchial epithelium) and alveoli. Particles can be either absorbed through the bronchial epithelium and enter systemic circulation or removed from the bronchial epithelium by mucociliary clearance (MC) and then absorbed in the gastrointestinal tract (GIT). Absorption pathway: black arrows; metabolization and excretion: blue arrows.

Biological effects start once particles get in contact with the respiratory epithelium, where they may be absorbed and enter systemic circulation or may be subjected to mucociliary clearance (Figure 2). Mucociliary clearance is the mechanism by which cilia of the bronchial epithelial cells remove particles trapped in mucus from the airways. The particles are transported towards the oral cavity, where they can be swallowed and taken up by the oral route. Alternatively, particles may be ingested by macrophages located at the air-blood barrier or be metabolized. The extent, to which these processes occur, is size-dependent.

Experimental methods to estimate the health risk by inhalation exposure to NPs, therefore, should address passage through respiratory barriers and effects on different cell populations at this barrier.

While transcellular and paracellular uptake of conventional compounds can be relatively well predicted from lipophilicity and molecular weight, the physiological behavior of NPs is more variable due to a higher number of influencing parameters, such as size, aggregation, shape, material, crystallinity, surface charge, and surface hydrophilicity. The small size allows NPs to reach the periphery of the lung, where gas exchange takes place. The interaction of NPs with the respiratory system is influenced by impaction, sedimentation, and diffusion. NPs are able to transmigrate the alveolar epithelium and enter systemic circulation, as well as connective and lymphatic tissue of the lung. ^99m^Technetium-labeled carbon NPs translocated to other organs in humans [16], while iridium and carbonaceous NPs showed only a low degree of translocation [17–19]. Crossing of the air-blood barrier by NPs in animals (rats and hamster) has been reported in several studies [20,21].

Methods to assess biological hazard of NPs at the air-blood barrier are the focus of this review. In addition, the combination of biological data with deposition data obtained by computer modeling for a realistic risk assessment of humans by exposure to inhaled NPs will be addressed.

## 2. *In Vitro* Systems

### 2.1. Cellular Models for NP Exposure

**Figure 3.**
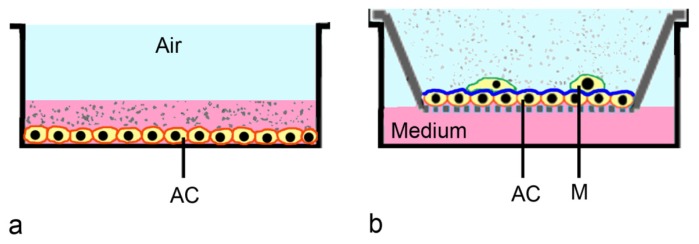
Exposure of alveolar cells in submerged culture and exposed to nanoparticle suspensions (**a**), and cultured in air-liquid interface exposed to nanoparticle-loaded aerosol (**b**). (**a**) Alveolar cells (AC) cultured in submersed culture usually do not differentiate and lack mucus or surfactant. NPs suspended in medium often form aggregates; (**b**) (co-culture shown): Alveolar cells cultured on transwells in the air-liquid interface produce surfactant (blue). NPs in aerosols usually form smaller aggregates than nanoparticles in suspensions. For further refinement of the model, co-culture with macrophages (M) on top of the epithelial cells (**b**) can be used.

In regulatory screening of drugs, cytotoxicity screening is used as initial step for the identification of hazard. A hazard is any source of potential damage, harm or adverse health effects on something or someone under certain conditions. This definition is often used when fixing threshold limit values in the work place. Regulatory cytotoxicity testing uses adherent cells cultured on plates and exposed to compounds suspended in medium (Figure 3a). This conventional set-up has some limitations for testing of respiratory cells, because in their physiological environment these cells are supplied with nutrients only from the basal side, while the apical pole of the cell faces the air. Therefore, cells should be cultured in a similar way prior to the exposure. Secondly, instead of using suspensions, NPs should be applied as aerosol. Deposition in submersed cultures is driven by Brownian diffusion and agglomeration and, therefore, is greatly different from deposition in the lung [22].

Due to the concern that cell culture systems could not represent the multicellular organism, toxicity testing was traditionally performed *in vivo*. With the possibility to use complex and physiologically relevant *in vitro* models based on human cells, *in vitro* testing has gained more popularity. Main advantages of *in vitro* testing are lack of concerns regarding cross-species correlation, ethical concerns, and economic constraints. *In vitro* models help in the understanding of toxicity mechanisms, although still some concerns regarding *in vitro* to *in vivo* correlations remain [23].

The lung tissue is composed of 40 different cell types [24] and it is not feasible to establish a single model containing all these cell types. Therefore, region-specific models have been developed representing one for the conducting zone of the lung (large airways, bronchioli) and another for the gas-exchange or respiratory zone (air-blood barrier, alveoli). An additional requirement is that these models should present the possibility for aerosol deposition. Refined multi-cellular 3D models, like for instance the one developed by Mondrinos *et al*. [25], express many physiological markers of the human lung, but they are not suitable for toxicity testing of inhaled NPs because they do not allow exposure to aerosols.

*In vitro* systems representing the respiratory tract should contain mainly respiratory cells and cells of the immune system. In the alveoli, which can come in contact with NPs, alveolar type (AT)-I cells form the actual lining while AT-II cells have secretory function (production of surfactant) and serve as progenitors for the terminally differentiated AT-I cells [26]. AT-II cells also produce a spectrum of pro-inflammatory cytokines. Macrophages in the alveoli remove particles and pathogens down to a size of 0.26 μm [27]; smaller particles evade phagocytosis.

Physiological exposure systems for respiratory cells employ transwell cultures. In these cultures cells are cultured on an insert, which is placed into a culture well. Medium is supplied from the basal side only and cells can be exposed to an aerosol at the apical part (air-liquid interface, ALI culture, Figure 3b). Transwell cultures were first used for permeability studies of gastrointestinal cells like Caco-2 cells and later adapted to other cell types [28]. For respiratory cells, ALI conditions are needed to induce secretion of mucus in Calu-3 cells [29] and surfactant in A549 cells [30,31]. These layers are important for particle retention and displacement under *in vivo* conditions [32]. Exposure of cells cultured in the ALI in a static diffusion chamber has been used for the assessment of diesel exhaust particles [33]. This system was later adapted by the same group for testing of dynamic exposure at airflows of ≤50 mL/min [34].

Various cells have been used to model the epithelial barrier. Primary cells, which are directly isolated from the tissue, are not usually preferred because of their limited life span and variations in their quality. This variability is due to donor variations and quality in the preparation. Immortalized cells (cell lines) although less well differentiated than primary cells, are most often used for the assessment of general cellular effects (cytotoxicity) and permeation. A549 cells, derived from a human adenocarcinoma of the lung, are the most often used cell line for toxicity testing [35]. The cells show properties such as surfactant production and transport like AT-II cells *in vivo*, secrete cytokines, and perform phase I and phase II xenobiotic biotransformation similar to lung tissue [36]. Although very useful for toxicity testing, A549 cells are less suitable to assess permeation as they do not form tight intercellular junctions. For the assessment of the bronchial barrier, Calu-3, 16HBE14o-, and BEAS-2B cells are used most often. The HPV-E6/E7 and hTERT immortalized bronchial epithelial cell line NuLi-1 has been more recently employed as model for bronchial epithelial barrier [37]. Permeability values obtained with Calu-3 and with 16HBE14o- cells appear to possess a high predictive value for absorption in lungs for conventional substances [38]. Models for the alveolar barrier use either primary or immortalized AT-II cells or NCI-H441 cells in mono- or in co-culture. In co-culture models for the assessment of permeation, cytotoxicity and pro-inflammatory effects, alveolar epithelial cells are mostly combined with cells of the immune system (macrophages, dendritic cells, mast cells) or with endothelial cells. Disease-relevant co-culture models (obstructive lung diseases) may also include fibroblasts. In one system, epithelial cells with macrophages on top are cultured on one side of the membrane and dendritic cells on the other [39]. The co-culture model developed by Alfaro-Moreno *et al*. [40] consists of a (10:2:1) mixture of A549 + THP-1 (monocytes) + HMC-1 (mast cells) cells in the basal compartment and an insert containing EAhy926 endothelial cells. These cells, thereafter, were exposed with suspended particles. Most recently, a similar co-culture model, also containing A549 epithelial cells, HMC-1 mast cells, THP-1 monocytes and EAhy926 endothelial cells, was characterized and used for aerosol exposure [41]. In this model, the endothelial cells were seeded on the basal side of the transwell, while A549 + THP-1 and HMC-1 cells were located on the apical side of the membrane. This way, the construct could be cultured in ALI and exposed to aerosols. Another co-culture system uses MRC-5 fibroblasts embedded in a collagen matrix on transwell membrane. On this layer PBMC-derived dendritic cells and, subsequently, epithelial cells were seeded [42]. This culture was cultured in ALI for an additional seven days but not used for aerosol exposure. Advantages of co-culture systems are the better representation of the physiological environment and also the identification of cell-specific differences in NP uptake. Upon exposure of the tetraculture model consisting of A549 + THP-1 + HMC-1 + endothelial cells, for instance, only THP-1 cells ingested the 50 nm silica particles. Disease-relevant models also include mechanical factors. Epithelial and endothelial cells cultured on both sides of a chip can be subjected to mechanical stress by changes of vacuum [43]. Bronchioconstriction can be mimicked by a co-culture in ALI of IMR-90 fibroblasts in collagen gel covered by normal bronchial epithelial cells in a strain device. In this device, cells are subjected to cyclic compression for 72 h [44].

For the assessment of permeation of NPs, transwell membranes may pose a significant barrier. Even in the absence of cells, the passage of polystyrene particles through membranes with pore size of 0.4 μm is considerable hindered [45,46]. Membranes with larger pores, for instance 1 or 3 μm, produce more reliable results for NP permeation. On the other hand, not all cells are able to form sufficiently tight intercellular junctions on membranes with larger pores. Large cells, for instance MDCK cells, show similar transepithelial electrical resistance when cultured on membranes with 0.4 μm diameter or with 3 μm diameter [47].

### 2.2. Commercial in Vitro Co-Cultures

Establishment and maintenance of co-culture systems is laborious and requires standardized operation protocols, which cannot be realized in all institutions. Therefore, commercial systems are provided by companies, such as MatTek and Epithelix, for users lacking the resources to establish their own know-how. These co-culture models are based on bronchial epithelial cells obtained from hospitals or patients donations and are optimized for specific applications. Cells are well differentiated, they produce mucus and show beating cilia, and maintain this differentiation over a period of several months [48]. The cells retain their ability for detoxification and are, therefore, suitable for the assessment of sensitization, in addition to uptake and transport studies. Since constructs from healthy and from diseased individuals are available, the influence of toxicants on pre-disposed persons can also be evaluated.

### 2.3. ALI-Based Exposure Systems

**Figure 4.**
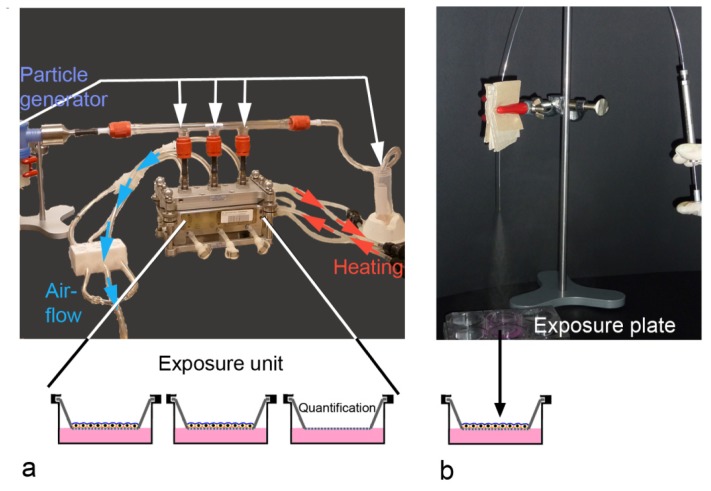
Set-up for aerosol-exposure at the air-liquid interface based on the VitroCell^®^ (Vitrocell Systems GmbH, Waldkirch, Germany) exposure system (**a**) and by manually generated aerosols (MicroSprayer^®^, **b**). (**a**) Cells are cultured on transwells and exposed in the compartments of the exposure unit, thermo stabilized by a water bath. Airflow generated by a vacuum pump provides a steady flow of the particle-loaded aerosol, generated in the particle generator (PARI BOY LC Sprint, PARI GmbH, Starnberg, Germany), over the cells. Part of the aerosol that passes the glass tube, is collected at the end of the glass tube and used for particle analysis in the aerosol. Particle deposition on cells is quantified in one compartment of the exposure unit; (**b**) Cells cultured on transwells are exposed in a separate exposure plate to one to three puffs of manually generated aerosol.

Many groups assessed the effects of environmental NPs (diesel exhaust, smoke) using either diffusion chambers or more advanced devices in static or dynamic exposure. Set-ups usually use exposures over 15–60 min, where the aerosol is generated and cells are exposed in a humid atmosphere at physiological temperature (37 °C) (Figure 4a, Vitrocell system shown). Particle deposition in most of the systems is driven by sedimentation and diffusion. Only few established systems, including Electrostatic Aerosol *in Vitro* Exposure system and CULTEX^®^ (Cultex^®^ Laboratories GmbH, Hannover, Germany) radial flow system, employ electrostatic precipitation. Voisin chamber [49,50], Minucell system [51,52], Nano Aerosol Chamber *in Vitro* Toxicity [53,54], Biological aerosol trigger [55], Air-Liquid Interface Cell Exposure system [56–58], and Electrostatic Aerosol *in Vitro* Exposure system [59,60] were developed by specific researcher groups. Other systems, such as CULTEX^®^ [61,62], CULTEX^®^ RFS, and VITROCELL^®^ [63], are commercially available. ALI-based exposure systems have been used for volatile organic compounds, copper NPs, carbon NPs, zinc oxide NPs, gold NPs, polystyrene NPs, cerium oxide NPs, and laser printer emission particles [33,51,64–66]. Quantification of the deposed aerosols is essential because aerosols or NPs contained in the aerosol may be retained by components of the exposure systems.

If accumulation of aerosol over time is not in the focus of interest, cells can be exposed with puffs of manually generated aerosols in plates (Figure 4b). MicroSprayer^®^ Aerosolizer (Penn-Century Inc., Glenside, PA, USA), a device commonly used for oropharyngeal exposure of rodents with NP suspensions, has been established for the assessment of polystyrene NPs [67]. Dose control upon delivery by the MicroSprayer^®^ (Penn-Century Inc., Glenside, PA, USA), is better and higher particle concentrations, independent from the material, can be delivered. For both, conventional substances and for NPs, delivery of the aerosolized material to the cells ranges between 25%–30% [68]. Multiple dosing is possible but generally limited by the amount of liquid from the aerosol delivered to the cells. This limitation could be avoided by the use of Dry Powder Insufflators™ (Penn-Century Inc., Glenside, PA, USA), from the same company, which generate aerosols from powders. A problem of manually generated aerosol could be shear stress generated by high air-flow rates through the sprayer.

For correlation of biological effects to the applied dose, analysis and quantification of the deposited amount of NPs is important. Deposition rates have been indicated in different units, mostly as μg/area/time or as % of the applied dose. Instead of gravitational measurements, quantification of indicator dyes loaded on the NPs may be used for calculation of deposition. Comparison of deposition efficacy between different exposure systems is complicated by differences in particle material and agglomeration kinetics. Deposition of polystyrene particles, for instance is markedly lower than that of carbon nanotubes [68]. In general, deposition efficacy of ALI exposure systems were reported as 0.03% for polystyrene particles in the Vitrocell system [68], 2% for carbonaceous NPs in the Minucell system [52], 9.48% for smoke particles in the CULTEX RFS system [69], and 8.81% for carbon nanotubes in the Vitrocell system [68]. When electrical charging and precipitation is used, deposition for polystyrene particles in the NACIVT system increases to 30% [54]. These relatively high deposition rates are also achieved when the MicroSprayer is used for exposure [68]. In addition to deposition, analysis should include particle size and chemical analysis.

## 3. *Ex Vivo* Systems

*Ex vivo* systems are relatively rarely used in the study of NPs. This is mainly due to technical difficulties in preparation and maintenance of isolated lungs. These difficulties, together with the limited life span of the isolated tissue, may be reasons for the rare application of this approach, compared to *in vitro* and *in vivo* exposures. Isolated perfused lung models from rats, guinea pigs, and rabbits have been established [70] where heart, lungs, and trachea are removed from the animal and placed into an artificial thoracic chamber. Trachea, pulmonary artery, and left atrium are cannulated and the perfusion medium enters via the pulmonary artery, flows through the pulmonary vasculature, and exits via a cannula in the left atrium, where samples can be drawn. The lungs are ventilated through the trachea with warm humidified air containing CO_2_ at negative pressure within the thoracic chamber. The thorax and the reservoir are kept at a temperature of 37 °C. Viability of the perfused lung can be maintained for two to three hours at 37 °C but thereafter, edema formation and cell death starts. When assessed in this *ex vivo* system, iridium NPs and polystyrene particles in sizes between 20 and 200 nm did not cross the air-blood barrier [71,72]. These findings are in contrast to *in vivo* data, where translocation of carbon NPs has been reported [72]. It can be presumed that the absence of lymph flow, hemodynamic factors and inflammatory cells in explanted lungs may cause the discrepant effects. Precision-cut lung slices are another type of *ex vivo* model that can be used for translocation and toxicity studies. Advantages compared to isolated lung preparations are the longer survival time (culture is possible for up to three days) and the testing of more samples (about 30 slices can be prepared from one rat lung) [73]. To prepare these slides, rodent trachea is filled with pre-warmed agarose-medium solution. After polymerization, sections of 200 μm in thickness were obtained from tissue cylinders. Solid lipid NPs were more cytotoxic in these lung slices than in A549 cells in conventional culture [74]. Lung slices were more sensitive to cobalt-ferrite NPs than NCI-H441 aleveolar cell but less sensitive than TK-6 lymphoblasts [75]. Although *ex vivo* models can better reproduce the complexity of the *in vivo* situation rather than *in vitro* models, deterioration of the explanted tissue and lack of hemodynamics can cause differences between *ex vivo* and *in vivo* data.

## 4. *In Vivo* Systems

*In vivo* testing has been initiated for a variety of NPs mainly exposed by the oral and dermal route. For inhalation, intratracheal instillation was often employed because rodents, the commonly used species for toxicity testing, are obligatory nose breathers and, therefore, not representative models for human inhalation exposure. Even when this limitation is accepted, it is financially and technically not possible to assess all currently known NPs *in vivo*. According to estimates, comprehensive long-term testing would cause costs of $1.18 billion and require 34–53 years [76]. Another negative aspect of animal experiments, obviously, is species difference, regarding kinetics and efficacy. The autophagy inhibitor 3-methyladenine, which reduced acute lung injury triggered by polyamidoamine dendrimers in mice, lacks clinical efficacy in humans due to reduced stability [77]. Different sensitivity to toxicants in rodent and human lungs is often explained by the much higher expression of metabolizing enzymes (mostly belonging to the CYP superfamily) in rodents’ lungs compared to human lungs [78].

On the other hand, many topics, such as retention of inhaled particles, afford long-term studies and cannot be performed *in vitro*. Inhaled NPs (≤100 nm) are retained in the body for longer periods; for instance, 75% of 100 nm carbon NPs were retained for more than 48 h in hamster lungs [79]. Reasons for the prolonged retention include deep penetration into the mucus or deposition in areas with reduced lung lining layer. In both cases, interaction with airway cells and likelihood of transmigration is increased. Surprisingly, not all particles retained in the lungs translocate to lymph nodes or enter the systemic circulation [80]. In inhalation studies with dogs, iridium NPs re-appeared on the epithelium, where macrophage-mediated clearance occurred and 90% of the inhaled NPs were recovered in the brochioalveolar lavage [81]. The capacity of macrophages to ingest particles is lower for NPs than for microparticles: 0.1% of TiO_2_ NPs compared to 87% of microparticles were internalized by macrophages after 24 h [82]. After 24 h only 1.7% of macrophages in BAL contained NPs compared to 12%–15% with microparticles [83]. It was concluded that at least during the first 24 h NPs could bypass phagocytosis by macrophages and interact with the epithelial barrier [79].

### 4.1. Inhalation Exposure Models

**Figure 5.**
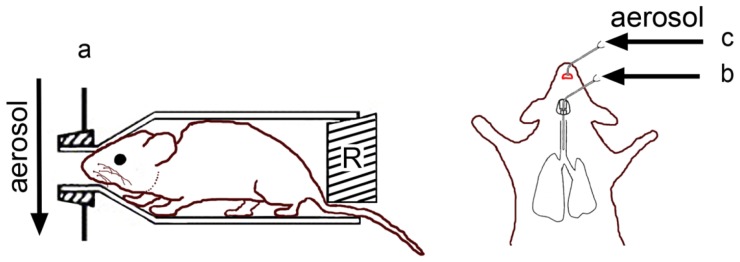
Exposure of rodents to aerosols. (a) nose-only exposure. The restraint (R) prevents loss of aerosol by leakage around the animal. The small opening at the bottom allows temperature regulation of the animal through the tail; Intratracheal instillation (b) and oropharyngeal aspiration (c) uses commercial or self-designed syringes for manual application of aerosol.

Common routes of inhalation exposure are whole-body exposure, nose/head-only exposure or lung-only exposure (intratracheal instillation/inhalation) ([84], Figure 5). The choice for the exposure technique is determined by availability of the testing material (whole body exposure needs high amounts of material), technical expertise of the personnel (intratracheal instillation is technically demanding), and the duration of the exposure (except for whole body exposure, anesthesia/sedation is needed, which is not tolerated by the animal for prolonged time periods). Dose-control is best with intratracheal instillation but this technique can cause local tissue damage and uneven distribution of the test substance in the lung. Dose per animal can be less well determined in chambers, where animals are housed together. An overview on the respective advantages and disadvantages is presented in Table 1. Some studies demonstrate the great impact of the exposure technique on the obtained results. In mice, inhaled single-walled carbon nanotubes elicited a greater effect than instilled particles [85], while the opposite was found for titanium dioxide NPs [86]. However, another study obtained similar results for both exposure techniques [87].

### 4.2. Whole-Body Exposure

In this type of exposure filtered compressed air is used to generate the aerosol, which then is heated and added to dry filtered room air. Before the aerosol is guided through the exposure chamber, a charge neutralizer is positioned to decrease electrostatic interaction with the chamber. At one part of the exposure chamber, the aerosol is collected for size determination and composition [88]. The requirements for aerosol generation are particularly high for this type of exposure. The aerosols in the chamber housing the animals should have a steady concentration of aerosol over the entire exposure time. Moreover, the aerosol should be free of contaminants and present a stable size distribution. The latter requirement is a particular challenge for NP-containing aerosols because NPs tend to agglomerate and form large agglomerates that cannot be broken up [89]. The doses animals receive can be highly variable. This variety can be due to the contribution of other routes of exposure (e.g., mouth, eyes); for instance, 60%–80% of the material deposited on the pelts of rats during the exposure is ingested and oral uptake contributes to the exposure [90]. In addition, the animals can avoid exposure by huddling together or burying their noses in corners of cages or in the fur of another animal.

### 4.3. Nose/Head Only Exposure

Compared to whole-body exposure, the chamber that holds the animal is very small. Aerosols are usually generated in one chamber for all exposed animals. For rodents it is usually a tube attached in a way that a hole or extension of the aerosol-producing chamber directs the atmosphere towards the animal’s nose (Figure 5a). At the back end of the tube a restraint is positioned to prevent the animal from backing out. The restraint can seal the back end of the tube completely and in this way prevent leakage of test air around the animal completely. To prevent overproduction of moisture and heat in the tube, the restraint can also only partially close the back end of the tube [91]. For mice and rats, for instance, an open restraint is preferred to facilitate heat loss via the tail and avoid overheating of the animal. For exposure times >1 h, additional cooling is usually advised [92]. The small chamber hinders animal movement and may cause discomfort. Younger animals may attempt to turn to escape from the tube, which bears the danger of suffocation [38]. Another problem is ventilation. If the flow through each port approaches the minute ventilation of the animal, the animal will rebreathe its exhaled atmosphere, carbon dioxide concentrations may increase and oxygen supply decrease. Eventually, the animal may suffocate. To prevent this, minimum flow through the nose-only chamber of 2.5 times the animal’s minute volume is recommended.

### 4.4. Lung-Only Exposure

Intratracheal instillation is performed by inserting a delivering device into the trachea and projecting its tip close to the bifurcation of the trachea (Figure 5b). Alternatively, the test aerosol may also be delivered by oropharyngeal intubation, where small animal laryngoscopes enable correct insertion of the delivery device. Devices in standard length and in custom sizes (Penn Century Inc., Glenside, PA, USA) are available for delivery of NP-loaded liquid aerosols [93] and from powders [94]. When coupled to a ventilator, a nebulization catheter can deliver a pulse-timed spray dosing delivery to the lung [95]. Oropharyngeal aspiration is even less invasive because a small volume of material is placed at the base of the tongue (Figure 5c). During inspiration by the animal the material is aspirated and distributes in the lung. This method was able to distribute polystyrene NPs and beryllium oxide particles throughout the lung [96]. Lung-only exposure may lead to artificial results by bypassing nose and defensive reflexes and may cause organ damage by dehydration of the trachea.

Historically, partial lung exposure was also used, where the test substance was injected in one lobe, while another lobe served as control. Anesthesia and precise placement of the catheters afford a great degree of technical skill. Although the applied dose is well-defined, non-physiological distribution within the lung may occur after initial placement [84].

### 4.5. Limitations of in Vivo Systems

Despite the established role of animal experimentation and advantages related to this kind of experiments, specific limitations apply for the testing of inhaled NPs.

#### 4.5.1. Interspecies Differences in Lung Physiology

Due to ethical issues and experimental costs, dogs and primates, showing the highest similarity to the human respiratory system, are rarely used for toxicity studies. Rats have been traditionally used for chemical toxicity testing and are also the most often used species for NP testing. Mice are interesting as around 2000 different strains of mice with carefully controlled genetics are available to study the influence on genetic variations on pathologies. Other small mammals are mostly used for specific research topics. Guinea pigs were used for sensitization to inhaled antigens since their airways show similar sensitivity to mediators as human airways, while rodent lungs are less sensitive. Great differences in lung parameters are seen between laboratory species and humans (Table 2). Extremes are tidal volume and respiratory rate of mice with 0.15 mL and 175 breaths/min, respectively. This compares with 500 mL and 15 breaths/min in healthy 70 kg humans [97,98]. Syrian hamster lungs have been mainly used for carcinogenesis and chronic respiratory studies.

Rats lungs also present prominent differences to human airways, particularly relevant to testing of particles [99]. While terminal bronchioles in rats measure 0.2 mm in diameter and 0.35 mm in length, they are 0.6 mm wide and 1.68 mm long in humans [100]. Particle deposition is minimal for sizes <0.5 μm in rats and humans but is maximal for 1 μm particles in rats and in sizes of 2–4 μm for humans [101]. Pulmonary deposition is, therefore, much higher for particles between 2–3 μm in humans than in rats (for human mouth breathing: ~50%, for human nasal breathing: ~25% and for rat (nasal) breathing: ~5%). In contrast to the extrathoracic (nasopharyngeal) deposition, the tracheobronchial and pulmonary (lobar) deposition fractions are practically insensitive to the change in aerodynamic diameter across the “respirable” size range of 1–5 μm. It is clear therefore, that lung-regional distribution can be altered little by changes in aerodynamic diameter in such animal models, a situation different from human inhalation. Mucociliary clearance velocities are higher in rats than in humans. While 10%–15% of particles (0.1–7 μm) deposited in the human bronchial tree are still detectable after 24 h, particles deposited in the rat bronchial tree are cleared after 6–8 h [102]. The delayed clearance appears to be due to the preferentially more peripheral deposition of particles in the human lung compared to a more central deposition in the rat lung. Mucus velocities decrease with decreasing diameter of the airways in both species and, as a consequence, small airways have a slower clearance.

Pulmonary studies in mice are problematic because not only pulmonal application of drugs but also measurement of lung function (flow, volume, and transpulmonary pressure) is technically challenging. They are less suitable than rats because differences in lung anatomy to humans are more pronounced than between rats and humans. In all these species the right lung has more lobes than the left lung. This asymmetry, however, is more pronounced in the laboratory rodents, where the right lung has four lobes and the left lung consists of only one lobe. The left lung of all small laboratory animals (mice, rats, hamsters) is not divided into lobes; only the larger laboratory animals, such as guinea pigs and rabbits, similar to humans, have left lungs with two lobes [103]. Additional differences are seen in the anatomy of peripheral airways and the interdigitation of conducting airways and gas-exchange regions. Respiratory bronchioles are extensive in cat, dog, sheep, monkey, and humans, and minimal in mouse, rat, hamster, rabbit, pig, cow, and horse. The combination of differences in the extent of respiratory bronchioles, in acinar size (in the order of 200 times), and in air-blood barrier thickness may account for the different sensitivity to inhaled toxicants between species. For instance, murine alveoli are very small with mean linear intercept of 80 μm, compared to 100 μm for rats and 210 μm in humans, and the air-blood barrier measures 0.32 μm in mice, 0.38 μm in rats, and 0.62 μm in humans [104]. Hamster lungs are similar to rodent lungs.

#### 4.5.2. Species-Specific Reaction to Particulates

The canine respiratory system presents many similarities to that of humans. Exposure to cigarette smoke inhalation produces clinical and histopathological changes similar to COPD and emphysema in humans [105]. Canine thoracic deposition for Co_3_O_4_ particles is not significantly different from that observed in the human respiratory tract rising from 12% to 35% of the inhaled particles of 0.7 to 3.7 μm aeodynamic diameter [106]. A percentage of 32% of 0.02 μm large particles and 25% of 0.1 μm large particles were deposited in canine lungs [107]. Based on the similarity between human and beagle lungs shown for larger particles, similar deposition rates are also expected in humans.

Despite differences between that rat and human respiratory systems, the rat is still most often used for toxicity testing of inhaled substances. Many studies identified differences in the reaction to particulates between humans and rats. Human AT-II cells proliferate as reaction to dust exposure to a much higher extent than rat AT-II cells, also smooth muscle hyperplasia as a reaction to smoke and mineral dusts is more pronounced in humans [108]. Silicates induce granuloma formation in both species but rodent lesions are more cellular and less fibrotic than those in humans. Profound remodeling as a reaction to asbestos and other fibrous minerals is seen only in humans. Although small airways in rodents have a different architecture and lack respiratory bronchioles, pathologic features of the small airways leading to the acini after exposure to dust are similar to that of humans. Some features developing after chronic exposure, such as emphysema, are difficult to detect in rats due to their short life span. Accumulation of dust is seen in the interstitial tissue in humans and intraluminarly in rats [109]. It is likely that the different accumulation patterns are the dominant reason for the observed differences in cellular responses. While granuloma formation was common in rats, fibrosis was the predominant response in humans. Acute intraluminal inflammatory and degenerative changes to inhaled fibrogenic and non-fibrogenic dust were more severe in rats than in humans. In humans only dusts that initiated epithelial hyperplasia were associated with lung cancer, while in rats all dusts induced epithelial neoplasia. The different reaction pattern can explain the differences in carcinogenicity between humans and rats [110].

## 5. *In Silico* Modeling

Due to the high permeability of the alveolar barrier, particle deposition is a decisive factor in absorption of inhaled drugs. Deposition is terminated when the particles get into contact with the airway wall. In addition to physical models for determining particle deposition in the human body, for instance the Mouth Throat Model, computational simulations have been used to estimate deposition of NPs in human lungs [111].

### 5.1. Mechanisms of Deposition

**Figure 6.**
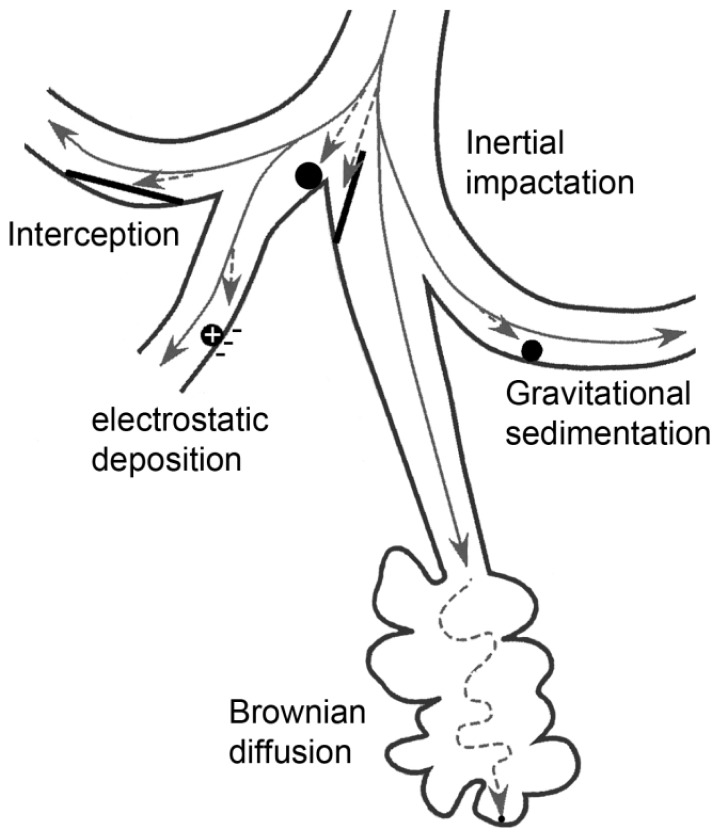
Particle parameters important for deposition in the whole lung. Large particles are subjected to inertial impactation, preferentially in large airways, smaller particles deposit by gravitational sedimentation, and small particles in the alveoli by diffusion. Electrostatic deposition is seen for charged particles and interception for fiber-shaped particles.

Generally, for the description of the respiratory deposition of particles, three components are required: geometrical model of the lung, aerodynamic characteristics, and particle behavior [112–114]. Depending on the aerodynamic diameter (AD) of inhaled particles, taking their shape and density into account, five deposition mechanisms are described: (1) inertial impaction; (2) sedimentation; (3) diffusion; (4) interception; and (5) electrostatic precipitation, which is related to particle charge (Figure 6). Inertial impaction is a significant deposition mechanism for particle with an AD larger than 2 μm. As these particles are too large to be able to stay in the moving air stream, they tend to inertially impact in the extrathoracic (ET) and upper tracheobronchial airways. For particles with an AD larger than 1 μm, gravity and sedimentation (gravitational sedimentation) are the dominant mechanisms for their deposition in the smaller conduction airways of the tracheobronchial tract. Particles with an AD 0.5 to 1 μm are subjected to Brownian diffusive deposition or will be exhaled. Interception deposition of particles depends on particle shape, for instance elongated particles like fibers are subjected to interception due to their length [115,116].

### 5.2. Deposition Models

Deposition models can be divided into two types, empirical models and mechanistic models.

The empirical models are based on mathematical equations fit to experimental data, and consider pathways in the respiratory tract as identical, having linear dimensions. Mechanistic models calculate the deposition rate in respiratory tract on the basis of a realistic description of lung structure and physiology, taking different breathing scenarios and parameters into account. These models are based on idealized descriptions of lung morphology and physiology and are based on computational fluid dynamics (CFD) or calculation of inhaled aerosol flow by considering the fate of a population of particles or an individual particle (Eulerian and Lagrangian models, respectively). Alternatively, deterministic or stochastic descriptions of the bronchial tree have been used [113,117–124].

Whereas the empirical models are used to calculate the deposition in the whole lung, the mechanistic models can be used for deposition either in the whole lung or in a specific region of the lung (local scale models). In this review only whole lung deposition models will be discussed. The local scale models are extensively reviewed elsewhere [113,121].

#### 5.2.1. Single Path Models

Empirical models were developed in the context of assessing the deposition of inhaled environmental and occupational pollutants. Such empirical models consider the human respiratory tract as a series of symmetrical anatomical compartments, through which the entire volume of the inhaled particles passes [114,121]. In such modeling, all pathways in the respiratory tract are identical and have equal linear dimensions [125,126]. Due to the symmetric branching, the deposition of inhaled particles is equal in each airway. The deposition correlations for the whole lung or for different compartments are provided based on fitting empirical data as a function of analytical parameters.

The first whole lung deposition model was developed by Findeisen [127]. This compartment model consisted of nine compartments, starting with the trachea and ending with alveolar sacs. Each compartment contained a number of parallel airway segments with identical diameter. Deposition mechanisms considered in the model were inertial impaction, sedimentation, Brownian diffusion, and interception. The efficiency of deposition by each mechanism in an airway segment was calculated using an approximate equation derived with simplifying assumptions. The deposition efficiencies calculated for various mechanisms were then summed to give the combined deposition efficiency. In this model, the airflow was assumed to have a uniform velocity profile in each airway segment and the entire volume of the inhaled aerosol was expected to reach every compartment.

This model has been further improved by different research groups, incorporating various refinements in morphometric components, aerodynamic characteristics in the lungs, and equations used in calculations for deposition efficiency. Yeh and Schum [126] developed a morphometric model consisting of the lung’s five lobes.

One of the most commonly used whole lung models for deposition and retention of inhaled radioactive particles has been developed by the International Commission on Radiological Protection (ICRP). In the latest version of ICRP [128] a five compartment model was used, comprising the anterior nose; the posterior nasal passages together with larynx and pharynx; the bronchial regions; the bronchioles and the alveolar region. The definition of these compartments is influenced by the specific clearance mechanisms associated with each compartment. For instance, deposition in the bronchial region and bronchioles is also defined as the fast-cleared fraction of deposition, and deposition in the alveolar region is also termed the slow-cleared fraction [113]. Empirical deposition equations derived from fitting experimental deposition data were used for ET region and considered the inertial impaction, sedimentation, and diffusion as deposition mechanisms.

Independently, the National Council on Radiation Protection and Measurements [129] has published another simulation using three compartment model, comprising (1) Naso-oro-pharyngo-laryngeal region; (2) Tracheobronchial region; and (3) Alveolar region. The major differences between the ICRP and NCRP models were in the modeling of tracheobronchial and alveolar regions by using different anatomical lung models and different deposition equations. Under the same conditions (particle size distribution, lung volume, and airflow), ICRP and NCRP models calculated similar deposition rates of particles in upper airway regions. However, the NCRP model gave higher deposition rates of particles smaller than 0.2 μm in the tracheobronchial tree and lower deposition in the alveolar region, which might be due to the fact that the convective mixing is not considered in NCRP model and the enhanced deposition because of branching bifurcation is not considered in ICRP model [130]. Detailed studies on enhanced deposition started in the 1970s, using three-dimensional airway models and improved using advanced CFD models [122,131–135]. The local deposition models are reviewed in detail by Longest and Holbrook [121].

Simple path models are attractive tools because of their geometric simplicity and ease of use. Moreover, they are in general agreement with *in vitro* fast and slow clearance fractions, representing the upper and lower airway depositions. The ICRP model in particular [128] finds wide application. This model is considered a standard model for routine inhalation dosimetry assessments and also integrated in software programs for calculating the deposition rate of pharmaceutical aerosols.

However, the efficiency of simple path models, especially for pharmaceutical aerosols, is limited due to the extremely simplified morphometry, physiology, and lung conditions, which is not suitable for calculating the particle deposition within a defined region [113,114,123].

#### 5.2.2. Multiple Path Models

Multi-path models have been developed to provide a more realistic lung-modeling than the single-path approach. In multi-path modeling, the asymmetry of the lung branching pattern and path variation of the bronchi have been taken into account.

The first approach in this field was the five-lobal model of Yeh and Schum [126], considering single-path models for each lobe instead of the generations of the tree structure. The morphometric models for the five lobes were based on the measurements of a silicone rubber cast of the human tracheobronchial tree [126,136]. The morphometric model of Raabe [136] was also the base for further approaches. Koblinger and Hofmann [137] used a Monte Carlo simulation to construct an airway geometry along each inhaled particle’s path by randomly selecting airway parameters from their frequency distributions and the correlations among them. While the airway geometry is selected randomly, particle deposition in individual airways is calculated analytically. Asymmetries of the airway bifurcations and airflow were included in the model. Deposition within each airway structure was calculated based on traditional correlations for sedimentation, impaction, and diffusion. The model was further developed [119] and showed good agreement with fast and slow clearance deposition fractions from *in vivo* studies. Further, Asgharian *et al*. [117] used the Monte Carlo approach for development of asymmetric bronchial geometry. In this approach, they used different lung structures including a typical-path model, five-lobe symmetric model, which was structurally different from the model of Yeh and Schum [126], and 10 stochastic lung models illustrating inter-subject variability among the human population. Using this model, lobar deposition was found to be significantly different between the five-lobe model and the stochastic lungs. A variation in deposition fraction of up to three fold was observed among various stochastic lungs, indicating that particle deposition within the lung is highly non-uniform. This model was further used for the investigation of the impact of asymmetric lung ventilation on lobar deposition [112]. Another application was the assessment of inter-subject variability in particle deposition in the respiratory tract and the deposition of nano-aerosols [138,139]. The primary impact of NPs was found in the pulmonary region for particles larger than 10 nm in diameter. Particles below 10 nm were removed from the inhaled air in the tracheobronchial region with little or no penetration into the pulmonary region. Good agreement was found between predicted depositions with measurements in the literature [138].

The advantage of multipath models over single path approach is the more realistic description of lung morphology and its asymmetric geometry. This results in more realistic determination of average deposition fractions. Moreover, the intra-and inter-subject variations in different parts of the lung can be determined more precisely. However, the validation of such models with *in vivo* data is only possible for total or regional deposition, but not at airway generation level. Application of “local scale models” based on CFD methods is an approach for overcoming the limitation of providing information on deposition patterns within airways or airway bifurcations. Computational models work best in the prediction of hydrophobic uncharged spherical particles in a static lung. In reality, particle size but also net particle charge, hydrophilicity, particle concentration, interception, thermophoresis (migration of the particle in a solution in response to a temperature gradient), and gas properties influence deposition. Biological parameters, such as tidal volume, breathing frequency, and airflow rates, markedly determine particle deposition [140]. Although several improvements have been undertaken to consider these parameters, still commonly used simulation models, such as ICRP and multiple path particle dosimetry model (MPPD), can simulate biological parameters only to a limited extent and, therefore, perform less well when simulating forced inhalation [141]. Simulation programs can also not include NP-specific effects, such as interaction with alveolar surfactant. Nanostructured SiO_2_ particles show a size-dependent reduction in surfactant activity [142], which implies that they can avoid clearance and are able to stay longer in the deposition zone.

## 6. Conclusions

Human health risk by NPs is judged highest for inhalation exposure because high permeability of the air-blood barrier allows fast uptake of particles. For a realistic assessment of particle deposition, uptake and biological effects, several models have to be combined to compensate for their respective limitations. *In vitro* systems lack the complexity of the entire organism but are important for identifying cellular mechanisms. Due to differences in breathing pattern, commonly used animal models cannot mimic human exposure but provide data on the fate of inhaled NPs in the body. *In silico* models could be useful for quantifying lung deposition and, when combined with permeation data obtained by cellular exposures and data from animal experiments, predict systemic effects of inhaled NPs.

## Figures and Tables

**Table 1.** Overview of advantages and disadvantages of *in vitro*, *ex vivo*, and *in vivo* studies.

Method	Application	Advantages	Limitations
*In vitro* techniques			

Conventional exposure (submersed)	High-throughput testing	Controlled dosing	Exposure of non-differentiated cells
	Initial screening for short-term effects	Easy to perform	Non-physiological exposure
		Efficient use of material	No information on permeation
			No complex (multicellular) response
			No long-term exposure

ALI (monoculture) + Suspension exposure	Mechanistic uptake and toxicity studies	Controlled dosing	Non-physiological exposure
		Study of differentiated cells	No complex (multicellular) response
		Efficient use of material	No long-term exposure
			Advanced technology

ALI (monoculture) + Aerosol exposure chamber	Mechanistic uptake and toxicity studies	Relatively controlled dosing	No complex (multicellular) response
	Permeation studies	Study of differentiated cells	No long-term exposure
		Efficient use of material	Complex exposure system
			Aerosol loss in the exposure system
			More complicated technology

ALI (mono/co-culture) + Aerosol spraying	Mechanistic uptake and toxicity studies	Controlled cellular dose	No long-term exposure
	Permeation studies	Study of differentiated cells	Potential shear stress of the cells
		Efficient use of material	More complicated technology

ALI (co-culture) + Aerosol exposure chamber	Absorption studies	Controlled dosing	Technically demanding
		Efficient use of material	No long-term exposure
		Study on several cell types	Aerosol loss in the exposure system
			Limited complex (multicellular) response

*Ex-vivo* techniques			

Isolated perfused lung	Absorption studies	Relatively controlled dosing	Technically demanding
		Complex (multicellular) response	Short observation time
		Physiological exposure	
		Efficient use of material	

Precision-cut lung slices	Toxicity studies	Controlled cellular dose	Non-physiological exposure
		Complex (multicellular) response	Short observation time
		Efficient use of material	

*In-vivo* techniques			

Whole-body exposure	ADME studies	Physiological way of exposure	Large amount of material needed
	Short-term/long-term, single exposure and multiple exposure	No anesthesia or discomfort for animals	Dose not well defined
		Complex (multicellular) response	

Nose/head only exposure	ADME studies	Relatively physiological way of exposure	Slight discomfort for animals
	Short-term/long-term, single exposure and multiple exposure	Not invasive, no anesthesia	Inexact dose control
		Complex (multicellular) response	

Intratracheal instillation	ADME studies	Direct dosing to lungs	Non-physiological exposure
	Short-term, single dose exposure	Complex (multicellular) response	Anesthesia needed
			No repeated dosing
			Tissue injury
			Labor intensive

Oropharyngeal instillation	ADME studies	Direct dosing to lungs	Non-physiological exposure
	Short-term, single dose exposure	Intubation not required	No repeated dosing
		Complex (multicellular) response	Labor intensive

Oropharyngeal aspiration	ADME studies	Direct dosing to lungs	Non-physiological exposure
	Short-term, single dose exposure	No intubation required	Potential aspiration of oral content into lungs
		Complex (multicellular) response	No repeated dosing
			Labor intensive

**Table 2.** Comparison of physiological lung parameters between laboratory animals and humans.

Species	Breath rate (resting, per minute)	Tidal volume (mL)	Total lung capacity (mL)
Rat	85	1	10
Mouse	163	0.15	1
Hamster	30	1	7
Guinea pig	84	1.7	23
Human	15	500	6000
